# Anterior Knee Unloader Brace Prescription Yields Moderate Compliance Rates and Improved Clinical Function in Patients with Patellofemoral Pain

**DOI:** 10.1002/ars2.70039

**Published:** 2026-06-15

**Authors:** Andrew R. Phillips, Thomas Moran, Kofi K. Acheampong, Stephanie A. Boden, Erik C. Haneberg, William D. Bugbee, John P. Fulkerson, Simon Goertz, John G. Lane, Sabrina M. Strickland, Adam B. Yanke

**Affiliations:** ^1^ Rush University Medical Center Chicago Illinois U.S.A.; ^2^ Division of Orthopaedic Surgery Scripps Clinic La Jolla California U.S.A.; ^3^ Department of Rehabilitation and Orthopedics, Yale School of Medicine New Haven Connecticut U.S.A.; ^4^ Brigham and Women's Hospital Boston Massachusetts U.S.A.; ^5^ Department of Orthopaedic Surgery, University of California San Diego California U.S.A.; ^6^ Hospital for Special Surgery, Sports Medicine Institute New York New York U.S.A.

## Abstract

**Purpose:**

To determine clinical outcome changes and wear compliance rates associated with the use of a custom‐fit anterior knee unloader brace in a prospective cohort of patients with clinical symptoms derived from patellofemoral osteoarthritis (PFOA).

**Methods:**

Adults ≥ 18 years with PFOA or multicompartment OA with patellofemoral involvement were enrolled in this study. Participants received a custom‐fit Icarus Ascender OA Brace with instructions to wear it during daily activity for 4 weeks. Patient‐reported outcomes (PROs) were collected at the pre‐ and postbracing intervals. PROs were compared from pre‐ to postbracing time points. Improvement from moderate or severe symptoms prebracing to mild or no symptoms postbracing was analyzed.

**Results:**

One hundred thirteen patients (134 knees) completed pre‐ and postbrace surveys (age: 53.5 ± 13.1 years, 43.4% female, 48.7% left knee). Sixty‐two (56.9%) patients reported wearing the brace ≥ 4 times a week, with 37 (33.9%) reporting daily use. Knee Injury and Osteoarthritis Outcome Score, Joint Replacement (KOOS JR) improved 21.62 ± 14.47 points (*P* < .001), with improvements of 0.88 ± 1.09 points (*P* < .001) in stiffness, 4.91 ± 3.17 points (*P* < .001) in pain, and 2.46 ± 1.82 points (*P* < .001) in Activities of Daily Living (ADL) subscores. Visual Analog Scale (VAS) pain scores improved 4.04 ± 2.10 points (59.3 ± 23.2%, *P* < .001). 66.42% of knees exceeded minimum clinically important difference (MCID) for KOOS JR. MCID achievement was higher in patients reporting “moderate or worse” stiffness (75.24%), pain (77.66%), and functional limitation (78.72%) baseline KOOS JR subscores compared with those reporting “mild or no” stiffness (39.39%), pain (40.00%), and functional limitation (37.50%). Sixty‐two of 71 (87.32%) patients reported “moderate or worse” symptoms for stiffness, pain, and ADL subscores.

**Conclusions:**

Prescription of an anterior knee unloader brace in patients with PFOA or multicompartmental knee OA with patellofemoral involvement results in moderate compliance and improvement in pain and clinical function in the majority of patients at short‐term follow‐up.

**Level of Evidence:**

Level IV, therapeutic retrospective case series.

## Graphical abstract

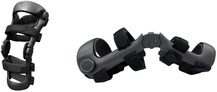

Knee osteoarthritis (OA) affects 14 million people in the US and is a leading cause of disability in adults worldwide.^1^, ^2^ OA can affect 1 or multiple knee compartments and result in pain and functional impairment. Nonoperative management is a viable treatment option for patients with uni‐ or tricompartmental knee OA, which includes the use of activity modification, oral anti‐inflammatory medications, physical therapy, various injection modalities, and bracing.^3^, ^4^ Bracing is a treatment option with a low safety risk profile that is recognized to improve symptoms and function in patients with OA.^5^, ^6^, ^7^, ^8^ Bracing options remain relatively understudied and underutilized in recent decades, however, partly due to low patient compliance with long‐term wear of traditional braces.^9^

Unloader braces have shown promise in improving pain and function in TF OA, particularly in the medial compartment,^10^ but their application in patellofemoral (PF) or multicompartment OA has been limited. The Ascender PF unloader brace (Icarus Medical, Charlottesville, VA) is a novel brace designed to address this gap in treatment. The brace produces joint distraction forces that assist the quadriceps during flexion, when PF joint forces are highest,^11^ thereby aiming to ‘unload’ the patellofemoral joint. Its design features a dynamic tensioning system that generates increasing torque in the brace hinge with increasing knee flexion activity, attempting to provide an ‘unloading’ effect in the PF compartment during knee flexion.^12^

In a preliminary prospective study of 64 patients who trialed the Ascender PF unloading brace, patients reported clinically and statistically significant improvements in pain, function, and stiffness, with high compliance rates.^12^ Therefore, the purpose of this study was to determine clinical outcome changes and wear compliance rates associated with the use of a custom‐fit anterior knee unloader brace in a prospective cohort of patients with clinical symptoms derived from patellofemoral OA (PFOA). We hypothesized that there would be significant improvements in clinical outcome scores after 4 weeks of using the custom‐fit PF unloader brace, with good brace wear compliance rates.

## METHODS

This study received IRB approval from Rush University Medical Center prior to data collection. Patients with PFOA or multicompartment OA involving the patellofemoral joint, who were over 18 years of age and ambulatory were enrolled in this study on a rolling basis over 3 years. Patients were not excluded from enrollment based upon attempt or failure of conservative treatment modalities. Enrolled patients were restricted from injection modalities during the six‐month period immediately preceding and during brace administration, they were not restricted from the utilization of other conservative treatment modalities. Clinical diagnosis of either PFOA or multicompartment OA involving the patellofemoral joint was made by fellowship‐trained, sports medicine surgeons (A.B.Y., W.D.B.). Presence of knee OA was confirmed by reviewing prescription and chart notes provided by the patient's prescribing physician. Upon enrollment, patients were presented with a prebrace survey, which included questions on patient demographics, intended use of the brace, treatment history, and current activity level. Additionally, patient‐reported outcome (PRO) surveys, including Knee Injury and Osteoarthritis Outcome Score for Joint Replacement (KOOS JR) and Visual Analog Scale (VAS) pain scale surveys were administered to collect baseline functional data. 4 weeks after brace delivery, patients were presented with a postbrace survey to capture KOOS JR scores and VAS pain scores data. Pre‐ and postbrace KOOS JR and VAS pain scores were compared to assess changes in subjective pain, function, and stability outcomes. Changes to patient activity level as a result of using the brace during the 4‐week period were also collected. All surveys were conducted in person or digitally through SurveySparrow (San Francisco, CA, USA).

### Brace Fitting

**FIGURE 1:**
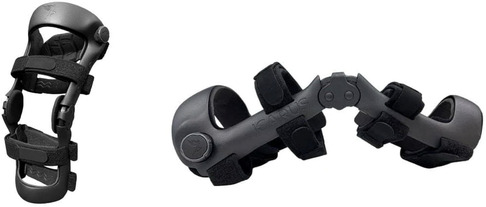
The Icarus Ascender brace.

Patients were scanned by a certified Icarus staff member using a mobile 3‐dimensional scanning phone application, and a custom brace was fabricated using 3D printing for the patient within 2 weeks of enrollment (Figure 1). The patient was then fit with the brace by Icarus staff or a partner clinic. Patients were provided education on donning and doffing the brace, operating the adjustable tensioning system, and received recommendations for daily use of the brace.

### Statistical Analysis

Given the presence of non‐normal data, nonparametric statistical analysis was performed. To compare pre‐ and postbracing KOOS JR, KOOS subscore, and VAS pain scores, paired Wilcoxon signed‐rank tests were used. In order to determine improvement from “moderate” or “severe” symptoms to “mild” or “no” symptoms, McNemar's test with continuity correction was performed. All statistical tests were 2‐tailed with a *P*‐value < .05 indicating significance. All statistical testing was performed in R Version 4.1.0 (R Core Team).

## RESULTS

Two hundred and forty‐nine patients were provided a brace during the study period and presented with the pre‐ and postbrace surveys. One hundred thirteen patients (134 knees, 45.4%) completed both the prebrace and 4‐week postbrace surveys. Patient demographics are listed in Table 1. Subjectively, 85 (75.2%) patients reported improvement in activity while wearing the unloader brace, with 14 (12.4%) noting significant improvement in activity. Additionally, patients stated they had improvement of pain in 125 knees (93.3%) during the bracing period.

**TABLE 1:** Demographics

**Variable**	**Mean ± Standard Deviation or N (%)**
Age	53.5 ± 13.1 years
Sex	
Male	64 (56.6%)
Female	49 (43.4%)
Laterality	
Right	37 (32.7%)
Left	55 (48.7%)
Bilateral	21 (18.6%)

### Compliance

**FIGURE 2:**
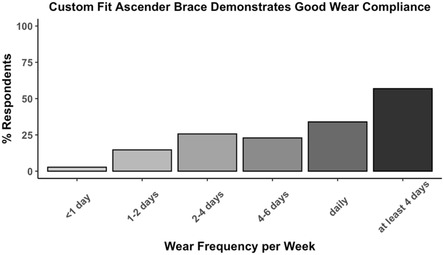
Brace wear compliance during the study period. Of the 109 respondents, 56.9% reported wearing their brace at least 4 days per week, with 33.9% wearing the brace daily.

Patients were asked how frequently they wore the brace during the study period. Sixty‐two respondents (56.9%) reported wearing the brace at least 4 times a week with 37 (33.9%) of patients reporting daily use. Only 3 patients reported less than 1 day per week use of the brace and no patient reported no use of the brace at all during the study time frame (Figure 2).

A majority of patients (55.8%) reported use during physical activities including exercise and sport. Other common uses for the brace included during daily tasks such as home or childcare (53.1%), work tasks (28.3%), and after the occurrence of knee pain (36.3%).

### Patient Reported Outcome Improvement

**FIGURE 3:**
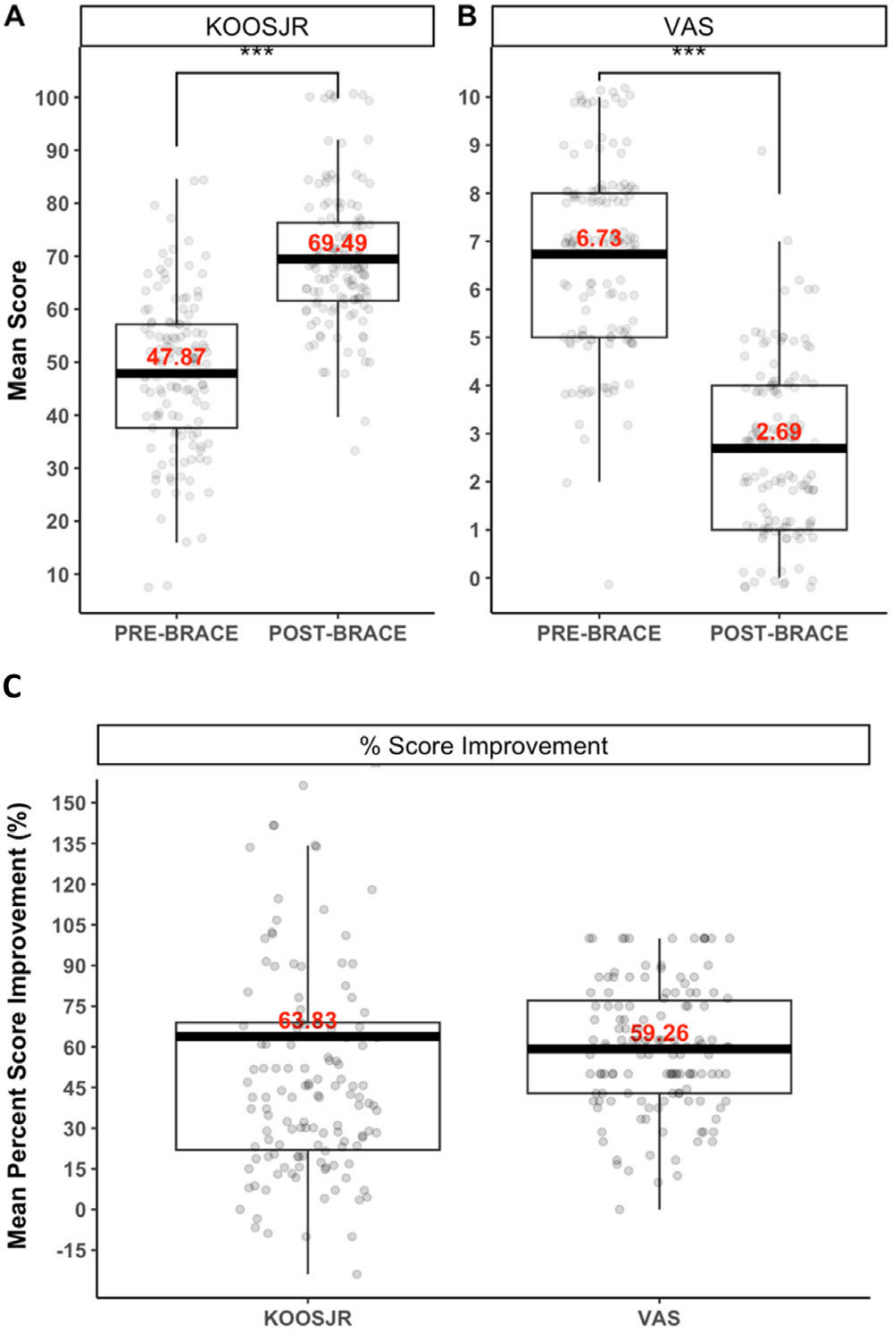
Custom‐fit Icarus Ascender brace use is associated with improved patient reported outcomes. At the end of the 4‐week study period, patients reported significant improvements in (A) KOOS JR (63.8% ± 92.0%) and (B) VAS (59.3 ± 23.2%) scores compared with their responses before brace use. (C) Mean score improvement in KOOS JR and VAS. Data are reported in mean (red). *** indicates *P*‐value < .001. (KOOS JR, Knee Osteoarthritis Outcomes Score for Joint Replacement; VAS, Visual Analog Score for Pain.)

KOOS JR, KOOS JR subscores, and VAS Pain scores showed significant improvement at the end of the bracing period (Table 2). KOOS JR scores improved by an average of 21.62 ± 14.47 points (63.8 ± 92.0%, *P* < .001), with stiffness subscore improvement of 0.88 ± 1.09 points (*P* < .001), pain subscore improvement of 4.91 ± 3.17 points (*P* < .001), and Activities of Daily Living (ADL) improvement of 2.46 ± 1.82 points (*P* < .001). VAS pain scores improved by a mean of 4.04 ± 2.10 points (59.3 ± 23.2%, *P* < .001) (Figure 3).

**TABLE 2:** Patient‐reported outcomes Scores Before and After Bracing Period

	**Prebrace**	**Postbrace**	***P*** **Value**
KOOS JR	47.87 ± 14.42	69.49 ± 12.83	**<.001** †
Stiffness subscore	2.16 ± 1.02	1.28 ± 0.90	**<.001** †
Pain subscore	8.92 ± 3.14	4.01 ± 2.59	**<.001** †
ADL subscore	4.43 ± 1.89	1.98 ± 1.52	**<.001** †
VAS pain	6.73 ± 1.95	2.69 ± 1.71	**<.001** †

*Note:* **Bold** indicates significant *P* value.

ADL, Activities of Daily Living; KOOS JR subscore; KOOS JR, Knee Osteoarthritis Outcomes Score for Joint Replacement; VAS Pain, Visual Analog Score for Pain.

† Wilcoxon test.

Patients who used the brace at least 4 times per week had greater improvement of the KOOS Stiffness subscore than those who used the brace less than 4 times per week (1.11 ± 1.07 vs 0.60 ± 1.06 points improved, *P* = .018). Additionally, these patients also reported more improvement in the KOOS ADL subscore (2.74 ± 1.99 vs 2.10 ± 1.54 points improved, *P* = .063) and VAS Pain score (4.26 ± 2.02 vs 3.77 ± 2.17 points improved, *P* = .079), but these differences did not reach statistical significance.

### Improvement in KOOS JR Subscores

Before the bracing period, 101 patients reported moderate or worse stiffness on the KOOS JR survey. Of these, 55 patients (54.5%) improved to mild or no stiffness after wearing the unloader brace (*P* < .001). Moderate or worse pain subscores were seen in 94 patients prior to application of the unloader brace, with 81 (86.2%) of these patients reporting improvement (*P* < .001). Similarly, moderate or worse functional subscores were improved to mild or no functional deficits in 77 (81.9%) of 94 patients (*P* < .001).

### Minimal Clinically Important Difference Achievement

At the end of the brace period, a total of 89/134 (66.42%) patients experienced improvements in KOOS JR scores that met or exceeded the previously established minimal clinically important difference threshold of 14.51.^13^ KOOS JR minimum clinically important difference (MCID) achievement was significantly higher among patients reporting ‘moderate or worse’ stiffness (75.24%), pain (77.66%), and functional limitation (78.72%) on their baseline KOOS JR survey compared with those reporting “mild or no” stiffness (39.39%), pain (40.00%), and functional limitation (37.50%) (Table 3). Among 71 patients who reported baseline “moderate or worse” symptoms for all 3 stiffness, pain, and ADL subscores, 62 (87.32%) achieved KOOS JR MCID compared to 5/16 (31.25%) of patients who reported “mild or no” KOOS JR stiffness, pain, or ADL symptoms (*P* < .001).

**TABLE 3:** Proportion of Patients Achieving Minimal Clinically Important Difference At the End of the Brace Period Grouped by their Pre‐brace Stiffness, Pain, and ADL Subscore Symptom Severity

**KOOS JR Subscore**	**Preoperative Symptom Severity Group**		***P*** **Value**
	*Moderate/Worse*	*Mild/No Symptoms*	
Stiffness	76/101 (75.24)	13/33 (39.39)	**<.001** †
Pain	73/94 (77.66)	16/40 (40.00)	**<.001** †
ADL	74/94 (78.72)	15/40 (37.50)	**<.001** †

*Note:* **Bold** indicates significant *P* value.

ADL, Activities of Daily Living KOOS JR subscore; KOOS JR, Knee Osteoarthritis Outcomes Score for Joint Replacement.

† Chi‐squared test.

## DISCUSSION

The most important finding of the current study is that patients with isolated patellofemoral arthritis or multicompartmental knee OA with patellofemoral involvement may experience improvement in pain and clinical function (KOOS JR, KOOS JR subscores, and VAS Pain scores) from the use of an anterior knee unloader brace at short‐term (4 week) follow‐up. Patients in the current study showed these outcomes with a prescription for short‐term brace wear and instruction for daily use, despite more moderate compliance and sporadic usage of this brace. Finally, MCID achievement with brace usage was significantly greater in patients characterized as having more significant knee dysfunction.

Multiple prior studies have shown clinical benefit with the use of off‐loading braces in the conservative management of knee OA.^14^ Although extensive research has been conducted regarding the effects of unloader braces in tibiofemoral compartment OA,^14^, ^15^, ^16^ there is a relative paucity of research looking specifically at off‐loading the patellofemoral joint in patients with patellofemoral OA. Patellofemoral OA represents a challenging clinical problem, as it can cause significant pain and functional disability with limited conservative management options. The patellofemoral off‐loader brace used in the current study is designed to provide dynamic unloading of the patellofemoral joint by assisting the quadriceps and therefore decreasing quadriceps activation and the transfer of load through the brace frame instead of the knee joint.^12^

The current study represents a more preliminary investigation into the clinical effectiveness of this novel anterior knee unloader brace. With 4 weeks of brace use, patients in this study were found to report significant improvements in both pain and stiffness, supporting the clinical utility in reduction of pain and improvement in function from the biomechanical effects purported by the brace design. Additionally, these differences in pre‐ to postbracing scores were found to have reached the MCID.^13^, ^17^ Patients that benefitted most from bracing were those with “moderate or worse” symptoms of dysfunction before bracing, as opposed to those with milder symptomatology before the initiation of bracing. This information is relevant in addressing patient expectations with use of the brace, as patients with more severe symptomatology may be counseled that they would be more likely to experience clinically significant improvement in symptoms with brace usage.

The current study also sought to provisionally examine brace compliance when standard instruction was provided to patients per typical clinical practice. Within this setting, patients showed moderate compliance with bracing and significant variation in brace wear. A dose‐dependent response to brace treatment existed in that a correlation between improvement in PROs and frequency of brace wear was identified. Within the current study, patients who used the brace at least 4 times per week had greater improvement in the KOOS Stiffness subscore than those who used the brace less than 4 times per week. Although similar trends in other patient reported outcome scores were seen between these groups, such as the KOOS ADL subscore and VAS pain score, they did not reach statistical significance. Although the differences in compliance are unable to be controlled for, the variation in brace usage is likely more representative of a real‐world setting and may better inform clinicians to expectations in use following brace prescription. From the current study, it remains unclear what accounted for the differences in compliance seen. Future investigation is necessary in order to determine if the differences observed are due to patient preference alone or representative of brace intolerance. Although there is utility in understanding the clinical benefit of bracing over this short‐term follow‐up, further longitudinal analysis is required to better understand brace tolerance and compliance over longer follow‐up. Future studies should evaluate the impact of this anterior knee unloader brace on other objective functional outcome measures, such as alterations in gait, analgesic medication requirements, ability to participate in desired activities, frequency of analgesic, corticosteroid, and biologic injections, and delays in operative management. Future studies should also involve comparative treatment in the setting of a randomized controlled trial to better evaluate the impact of this anterior knee unloader brace on pain and function.

### Limitations

The current study contains several limitations, mostly due to the nature of its design as a prospective case series. Therefore, patients were not randomized to treatment and no control group existed for comparison. There is also inherent risk of selection bias both in the form of which patients were offered bracing, which patients opted to undergo a trial of bracing, and which patients ultimately completed pre‐ and postbrace surveys. Specifically, just under half of enrolled patients completed pre‐ and postbrace surveys. This study is unable to account for this difference in response. There also exists some possibility for the introduction of various confounding variables. Although patients were restricted from injections during the period immediately preceding and during brace administration, they were not restricted from other conservative treatment modalities. Although differences in these treatments could impact the findings, they are representative of a real‐world setting in which patients employ multifactorial nonoperative treatments in order to improve pain and function from PF and multicompartmental OA. The study period was also brief, with outcomes collected at 4 weeks after initiation of bracing. Finally, the study was limited in that it only investigated patient reported outcome measures and did not look at other outcome measures such as frequency of injection or failure of conservative management with ultimate operative intervention.

## CONCLUSIONS

Prescription of an anterior knee unloader brace in patients with PFOA or multicompartmental knee OA with patellofemoral involvement results in moderate compliance and improvement in pain and clinical function in the majority of patients at short‐term follow‐up.

## DISCLOSURES

The authors (W.D.B., S.G., J.G.L., A.B.Y.) declare the following financial interests/personal relationships which may be considered as potential competing interests: W.D.B. reports a relationship with ICARUS Medical that includes: consulting or advisory and equity or stocks. S.G. reports a relationship with Icarus Medical Innovations that includes: stock or stock options. J.G.L. reports a relationship with Icarus Medical Innovations that includes: stock or stock options. A.B.Y. reports a relationship with ICARUS Medical that includes: equity or stocks. The other authors (A.R.P., T.M., K.K.A., S.A.B., E.C.H., J.P.F., S.M.S.) declare that they have no known competing financial interests or personal relationships that could have appeared to influence the work reported in this article.
